# Dynamic fluctuations in brain iron content during migraine attacks: insights from relaxometry and diffusion tensor imaging

**DOI:** 10.3389/fneur.2024.1422313

**Published:** 2024-12-20

**Authors:** Christoph Birkl, Vera Filippi, Ruth Steiger, Florian Frank, Stephanie Magnesius, Elke R. Gizewski, Gregor Broessner

**Affiliations:** ^1^Department of Neuroradiology, Medical University of Innsbruck, Innsbruck, Austria; ^2^Neuroimaging Research Core Facility, Medical University of Innsbruck, Innsbruck, Austria; ^3^Department of Neurology, Headache Outpatient Clinic, Medical University of Innsbruck, Innsbruck, Austria

**Keywords:** migraine cycle, pain processing, brain iron, R_2_^*^ relaxometry, quantitative MRI

## Abstract

**Background:**

There is evidence that iron metabolism may play a role in the underlying pathophysiological mechanism of migraine. Studies using $R_{2}^{*}$ (=1/$T_{2}^{*}$) relaxometry, a common MRI-based iron mapping technique, have reported increased $R_{2}^{*}$ values in various brain structures of migraineurs, indicating iron accumulation compared to healthy controls.

**Purpose:**

To investigate whether there are short-term changes in $R_{2}^{*}$ during a migraine attack.

**Population:**

26-year-old male patient diagnosed with episodic migraine with aura according to ICHD-3 criteria.

**Sequence:**

3 T, 64-channel head coil, for quantification of $R_{2}^{*}$ relaxation a multi-echo gradient echo (GRE) sequence with TE = 4.92, 9.84, 14.7, 19.6, 24.6 and 29.51 ms, TR = 35 ms, flip angle = 15°, and 0.9 × 0.9 × 0.9 mm^3^ isotropic resolution was used.

**Assessment:**

Quantitative MRI, including $R_{2}^{*}$ relaxometry and diffusion tensor imaging (DTI), was acquired from a migraine patient on 21 consecutive days, including migraine-free days and days with a migraine attack.

**Statistical test:**

Statistical analysis was performed using R, the Shapiro–Wilk test, the *t*-test and Mann Whitney *U* test, analysis of variance (ANOVA) or Kruskal–Wallis test, depending on the distribution of the data. *p*-value <0.05 was considered significant.

**Results:**

Significant difference in $R_{2}^{*}$ was found between the left and right hemispheres during a migraine attack. An increase in $R_{2}^{*}$ was observed in the left hemisphere, whereas in the right hemisphere $R_{2}^{*}$ was found to decrease. In the left cerebral white matter, $R_{2}^{*}$ increased by 1.8% (*p* = 0.021), in the right cerebral white matter, $R_{2}^{*}$ anisotropy decreased by 17% (*p* = 0.011) during a migraine attack.

**Data conclusion:**

Our study showed a decrease and increase in iron content during the migraine cycle. Furthermore, during a migraine attack, white matter iron content increased, accompanied by a decrease in anisotropic tissue components, suggesting additional changes in vascular components.

## Introduction

Migraine is a primary headache disorder typically characterized by recurrent attacks of disabling headache and associated with relevant personal and societal burden (1). The detailed pathophysiological mechanisms causing migraine are still elusive, however, there is evidence that central iron metabolization might play a role (2, 3). Evidence suggests that iron imbalance, particularly iron deficiency or overload, may be associated with migraines through mechanisms involving oxidative stress, neurotransmitter regulation, and vascular health (4, 5). Iron is a metabolically very active component. Some of the reasons for high iron levels in brainstem structures include overproduction of transferrin, increased iron uptake reflecting increased activity, and sequestered iron following cell damage regardless of the mechanism, abnormally high or low iron affects homeostasis and is a marker of altered function (5–7).

Various magnetic resonance imaging (MRI) methods enable measurement of iron *in vivo* in the human brain (8, 9). *R*_2_ (=1/*T*_2_) or $R_{2}^{*}$ (=1/$T_{2}^{*}$) relaxometry are one of the most commonly used MRI based iron mapping techniques, as there is a strong linear relationship between *R*_2_ and $R_{2}^{*}$ values with the underlying iron content in brain structures (10). Several MRI studies observed altered iron sensitive quantitative MRI measures in various brain structures of patients with migraine, indicating an iron accumulation compared to healthy controls (6, 7, 11–14). This increase in iron content was associated with pain processing and the frequency of migraine attacks (7, 11, 14).

Although, there is a strong correlation between $R_{2}^{*}$ and iron content in gray matter, several confounding factors, such as variations in tissue microstructure, myelin content and water content, exist which counteract the effect of iron on $R_{2}^{*}$ in white matter (15, 16). In white matter, the high amount of myelin leads to a strong confounding effect on $R_{2}^{*}$, as both, an increase in iron and an increase in myelin, leads to higher $R_{2}^{*}$ values and vice versa. Furthermore, $R_{2}^{*}$ in white matter is sensitive to the orientation of anisotropic tissue structures with respect to the *B*_0_ field of the MRI system (17, 18). There two main sources of $R_{2}^{*}$ anisotropy in the brain are (I) myelinated nerve fibers (17, 18) and (II) the anisotropic component of the vasculature (larger vessels tend to run in parallel with nerve fiber tracts) (19). However, the fiber orientation dependency of $R_{2}^{*}$ can be utilized to separate the effect of iron and anisotropic structures on $R_{2}^{*}$ in white matter. Therefore, $R_{2}^{*}$ is combined with the fiber angle, estimated using diffusion tensor imaging (DTI), within each white matter voxel to compute the fiber orientation independent (isotropic) and fiber orientation dependent (anisotropic) $R_{2}^{*}$ components (20, 21).

Although, there are many studies on structural MRI in patients with migraine (22, 23) there is limited data on MRI measurements during an acute migraine attack. Therefore, the aim of this study was to investigate if there are dynamic fluctuations in isotropic and anisotropic $R_{2}^{*}$, which can be related to tissue components, such as iron, in the brain during a migraine attack. To achieve this, we acquired, quantitative MRI, including $R_{2}^{*}$ relaxometry and DTI of a patient with migraine on 21 consecutive days, comparing migraine-free days and 2 days with an acute migraine attack.

## Materials and methods

A 26-year-old male patient diagnosed with episodic migraine with aura according to ICHD-3 (1) criteria since the age of 15 years participated in this study. He reported aura symptoms as mainly flashing lights lasting 15–20 min in the left visual field, mostly before the onset of the migraine headache. The participant had a history of 4 to 5 migraine attacks per month before entering the study. During the study the participant experienced two migraine attacks (on day 12 and day 16), which fulfilled the criteria for a migraine attack as defined by the ICHD-3 (1). The attacks lasted up to 24 h and were always localised on the left frontal side. Pain maxima ranged from 70–80 on a visual analogue pain scale of 0–100, with 100 representing maximum pain. Individual migraine attacks were always preceded by a prodrome, characterised mainly by tiredness and yawning. After the migraine attacks had subsided, a postdrome phase was clinically observed, characterised by fatigue and impaired concentration. During the 21 consecutive scanning days, the participant voluntarily decided not to take any preventive or acute medication and refrained from any other medication. The participant was a non-smoker, did not drink alcohol during the study period and maintained a constant daily routine prior to each measurement. The subject’s informed consent was obtained in accordance with the Declaration of Helsinki and approved by the Ethics Committee of the Medical University of Innsbruck. Written informed consent was obtained from the subject for the publication of any potentially identifiable images or data included in this article.

### Magnetic resonance imaging acquisition

MRI was performed at the same time on each day on a 3 T MR system (MAGNETOM Skyra, Siemens Healthineers, Erlangen, Germany) using a 64-channel head coil. The following sequences were acquired in this study: For structural overview and tissue segmentation, a 3D *T*_1_ weighted magnetization prepared rapid acquisition gradient echo (MPRAGE) sequence with echo time (TE) = 2.1 ms, repetition time (TR) = 1,690 ms, inversion time (TI) = 900 ms, flip angle = 8° and a 0.8 × 0.8 × 0.8 mm^3^ isotropic resolution. For the estimation of the white matter fiber angle *θ*, a diffusion weighted single-shot echo-planar imaging DTI sequence with TE = 92 ms, TR = 9,600 ms, flip angle = 90°, 30 isotropically distributed diffusion directions, *b*-value = 1,000 s/mm^2^, three images with *b*-value = 0 s/mm^2^ and a 2 × 2 × 2 mm^3^ isotropic resolution. For quantification of $R_{2}^{*}$ relaxation, a multi-echo gradient echo (GRE) sequence with TE = 4.92, 9.84, 14.7, 19.6, 24.6 and 29.51 ms, TR = 35 ms, flip angle = 15°, and 0.9 × 0.9 × 0.9 mm^3^ isotropic resolution.

### Image processing

$R_{2}^{*}$ maps were computed voxel by voxel assuming a mono-exponential relaxation using MATLAB 2019a (The MathWorks Inc., Natick, Massachusetts, United States). DTI data was analyzed with the FMRIB Software Library (FSL version 6.0.5.1) using FSL DTIFIT (24, 25) to calculate the diffusion tensor model and estimate the eigenvalues and eigenvectors. To correct for distortions induced by eddy currents and head motion FSL’s eddy_correct was used. The fiber angle *θ* was calculated as the angle between the first eigenvector and the direction of the main magnetic field *B*_0_ for each voxel, where *θ* = 0° represents fibers parallel to *B*_0_ and *θ* = 90° represents fibers perpendicular to *B*_0_. For plotting fiber orientation dependent $R_{2}^{*}$, the fiber angle *θ* was divided into 18 intervals of 5° and voxels from the entire white matter were pooled. $R_{2}^{*}$ anisotropy (Equation 1) (20) was calculated based on orientation dependent $R_{2}^{*}$ according to


(1) $$R_{2}^{*}\;\mathrm{ansiotropy}=\frac{\left(R_{2,\max}^{*}-R_{2,\min}^{*}\right)}{\left(R_{2,\max}^{*}+R_{2,\min}^{*}\right)}$$

where $R_{2,\max}^{*}$ represents the maximum and $R_{2,\min}^{*}$ the minimum value of $R_{2}^{*}$ as function of fiber angle.

*T*_1_ weighted images were used for automated segmentation of white matter, deep gray matter and cortical brain structures using FreeSurfer software (version 7.3.2). Automated tissue segmentation was performed independently on each day. A list of all segmented brain regions can be found in Table 1.

**Table 1:** Regional $R_{2}^{*}$ values averaged over all migraine-free days and migraine days.

	Hemisphere	$R_{2}^{*}$ (1/s)				Δ$R_{2}^{*}$ (%)	*p*-value
		Migraine-free		Migraine			
		Mean	SD	Mean	SD		
Thalamus	Left	20.6	0.4	20.6	0.1	0.0	0.947
	Right	20.7	0.5	20.4	0.5	−1.4	0.467
Caudate	Left	20.6	0.6	21.6	0.2	4.9	0.021
	Right	20.8	0.8	19.7	0.4	−5.3	0.114
Putamen	Left	23.3	0.4	23.0	0.8	−1.3	0.533
	Right	22.5	0.6	22.9	0.1	1.8	0.343
Pallidum	Left	31.4	1.2	32.0	0.6	1.9	0.467
	Right	31.1	0.7	31.6	0.1	1.6	0.316
Hippocampus	Left	17.0	0.4	17.7	0.1	4.1	0.063
	Right	17.1	0.4	16.8	0.5	−1.8	0.379
Amygdala	Left	16.0	0.7	15.2	0.1	−5.0	0.187
	Right	14.7	0.7	14.8	0.7	0.7	0.952
Accumbens-area	Left	17.3	1.6	16.9	1.8	−2.3	0.686
	Right	18.3	1.0	17.2	0.4	−6.0	0.191
Ventral DC	Left	23.0	0.5	24.3	0.3	5.7	0.011
	Right	22.0	0.7	21.0	0.5	−4.5	0.086
Cerebral-white-matter	Left	21.0	0.2	21.4	0.1	1.9	0.021
	Right	21.0	0.3	20.8	0.1	−1.0	0.286
Optic-chiasm		18.6	2.1	18.0	0.7	−3.2	0.590
CC-posterior		24.3	0.8	23.1	0.3	−4.9	0.057
CC-mid-posterior		20.5	1.0	20.5	0.2	0.0	0.686
CC-central		19.8	1.1	20.9	0.1	5.6	0.238
CC-mid-anterior		18.1	0.5	18.1	0.3	0.0	0.990
CC-anterior		21.9	1.7	20.9	0.6	−4.6	0.343
Bankssts	Left	18.1	0.4	18.2	0.1	0.6	0.857
	Right	17.7	0.6	17.2	0.4	−2.8	0.191
Caudal anterior cingulate	Left	14.9	1.1	15.6	0.5	4.7	0.316
	Right	15.0	0.6	14.8	0.1	−1.3	0.441
Caudal middle frontal	Left	16.7	0.2	17.0	0.2	1.8	0.095
	Right	16.6	0.3	16.1	0.1	−3.0	0.021
Cuneus	Left	21.3	0.7	21.0	0.6	−1.4	0.533
	Right	23.4	0.8	23.5	0.1	0.4	0.947
Entorhinal	Left	22.2	2.5	22.5	1.8	1.4	0.857
	Right	18.7	2.1	19.7	0.2	5.3	0.857
Fusiform	Left	21.2	0.4	21.6	0.5	1.9	0.267
	Right	20.5	0.5	20.1	0.1	−2.0	0.168
Inferior parietal	Left	17.6	0.4	17.7	0.5	0.6	0.771
	Right	18.1	0.4	17.3	0.5	−4.4	0.086
Inferior temporal	Left	24.3	0.8	24.7	0.2	1.6	0.590
	Right	24.3	1.0	24.4	0.5	0.4	0.952
Isthmus cingulate	Left	19.8	0.5	19.3	0.1	−2.5	0.211
	Right	19.3	0.9	18.4	0.2	−4.7	0.114
Lateral occipital	Left	20.8	0.4	20.5	0.2	−1.4	0.286
	Right	20.8	0.5	20.0	0.5	−3.8	0.057
Lateral orbitofrontal	Left	27.4	0.8	27.7	0.1	1.1	0.467
	Right	27.5	0.9	26.6	0.6	−3.3	0.152
Lingual	Left	20.4	0.7	20.1	0.2	−1.5	0.674
	Right	21.5	0.8	20.7	0.2	−3.7	0.152
Medial orbitofrontal	Left	23.2	0.8	23.8	1.0	2.6	0.442
	Right	21.4	1.1	20.2	1.1	−5.6	0.191
Middle temporal	Left	18.2	0.5	18.0	0.2	−1.1	0.467
	Right	19.5	0.4	19.5	0.2	0.0	0.732
Parahippocampal	Left	25.3	0.9	25.5	1.0	0.8	0.674
	Right	19.6	0.7	19.1	0.8	−2.6	0.467
Paracentral	Left	18.1	0.5	18.3	0.1	1.1	0.343
	Right	17.9	0.4	18.1	0.1	1.1	0.516
Pars opercularis	Left	16.9	0.3	16.6	0.1	−1.8	0.086
	Right	15.9	0.4	15.5	0.1	−2.5	0.191
Pars orbitalis	Left	18.5	0.6	18.0	0.9	−2.7	0.379
	Right	18.4	0.6	18.5	0.2	0.5	0.686
Pars triangularis	Left	17.3	0.5	16.9	0.2	−2.3	0.114
	Right	16.8	0.5	16.6	0.2	−1.2	0.674
Pericalcarine	Left	19.9	0.6	19.9	0.2	0.0	0.952
	Right	19.7	0.5	18.8	0.1	−4.6	0.047
Postcentral	Left	18.0	0.2	17.9	0.2	−0.6	0.442
	Right	17.6	0.3	17.3	0.1	−1.7	0.343
Posterior cingulate	Left	16.5	0.3	16.3	0.1	−1.2	0.316
	Right	16.4	0.5	16.4	0.1	0.0	0.771
Precentral	Left	17.7	0.3	17.7	0.3	0.0	0.952
	Right	17.5	0.2	17.3	0.1	−1.1	0.132
Precuneus	Left	17.9	0.3	18.1	0.3	1.1	0.442
	Right	17.7	0.6	17.4	0.4	−1.7	0.400
Rostral anterior cingulate	Left	14.8	1.1	15.2	0.1	2.7	0.442
	Right	14.3	0.9	13.8	0.3	−3.5	0.379
Rostral middle frontal	Left	17.3	0.3	16.9	0.3	−2.3	0.114
	Right	17.4	0.3	17.3	0.2	−0.6	0.771
Superior frontal	Left	15.9	0.6	16.1	0.2	1.3	0.533
	Right	15.6	0.1	15.3	0.1	−1.9	0.026
Superior parietal	Left	18.1	0.3	17.9	0.3	−1.1	0.400
	Right	18.1	0.4	17.8	0.4	−1.7	0.343
Superior temporal	Left	16.8	0.3	16.9	0.1	0.6	0.590
	Right	16.2	0.2	16.1	0.3	−0.6	0.573
Supramarginal	Left	17.5	0.3	17.7	0.2	1.1	0.379
	Right	17.4	0.3	17.0	0.5	−2.3	0.263
Frontal pole	Left	16.8	1.2	16.3	0.5	−3.0	0.533
	Right	16.3	0.9	16.1	0.7	−1.2	0.857
Temporal pole	Left	12.2	1.6	16.1	0.5	32.0	0.533
	Right	10.0	1.5	14.8	0.5	48.0	0.610
Transverse temporal	Left	18.1	0.6	18.6	0.2	2.8	0.126
	Right	17.0	0.4	16.7	0.3	−1.8	0.421
Insula	Left	16.2	0.7	16.3	0.8	0.6	0.990
	Right	15.6	0.5	16.1	0.3	3.2	0.263
Cerebellum-white-matter	Left	20.0	0.9	21.1	0.7	5.5	0.086
	Right	19.9	0.9	20.4	0.1	2.5	0.343
Cerebellum-cortex	Left	20.3	0.3	20.2	0.1	−0.5	0.933
	Right	20.2	0.7	19.3	0.3	−4.5	0.070

R^*^_2_ difference between migraine-free and migraine condition is given in percentage.

### Statistical analysis

Statistical analysis was performed using R (version 4.0.3, The R Foundation for Statistical Computing, Vienna, Austria). A Shapiro–Wilk test was used to test for normal distribution of the data. Depending on the data distribution, a *t*-test or Mann Whitney *U* test was used to assess differences of $R_{2}^{*}$ in various brain regions of the left and right hemisphere. To test if $R_{2}^{*}$ was different on days with migraine, an analysis of variance (ANOVA) or Kruskal–Wallis test was used depending on the distribution of the data. As post-hoc tests, pairwise *t*-test or Mann Whitney *U* test were applied. For *p*-value adjustment Bonferroni correction was used. We repeated the entire analysis, excluding days right after the migraine attack from the migraine-free days, to investigate if there are potential changes on these days. Study personnel was fully blinded regarding migraine status (migraine-free vs. migraine) until completed data acquisition and computation of the quantitative MRI parameter maps.

### Data availability

The transfer/disclosure of raw MRI data to 3rd party had not been approved in the ethical approval obtained for this study (issued by the local ethical committee of the Medical University of Innsbruck, EC Nr: 1324/2019).

## Results

### Radiological report regarding structural MRI sequences

Prominent focal veins were visualized on the left hemisphere, and asymmetry in the appearance of the cortical vessels was more prominent on the left side in the susceptibility weighted imaging (SWI). Enlarged and clustered perivascular spaces (PVS) are visualized in 3D *T*_1_ weighted images in the deep grey matter bilaterally, mildly accentuated on the left side. Accentuated PVS were also present in the supratentorial white matter, specifically in the centrum semiovale, with no clear side difference. PVS in this patient were considered radiological within normal ranges. No diffusion restrictions suggesting ischemic processes-were present and the cerebral fluid system was normal. Overall, structural MRI was without findings and considered radiological within normal range.

### Quantitative MRI analysis

**Figure 1:**
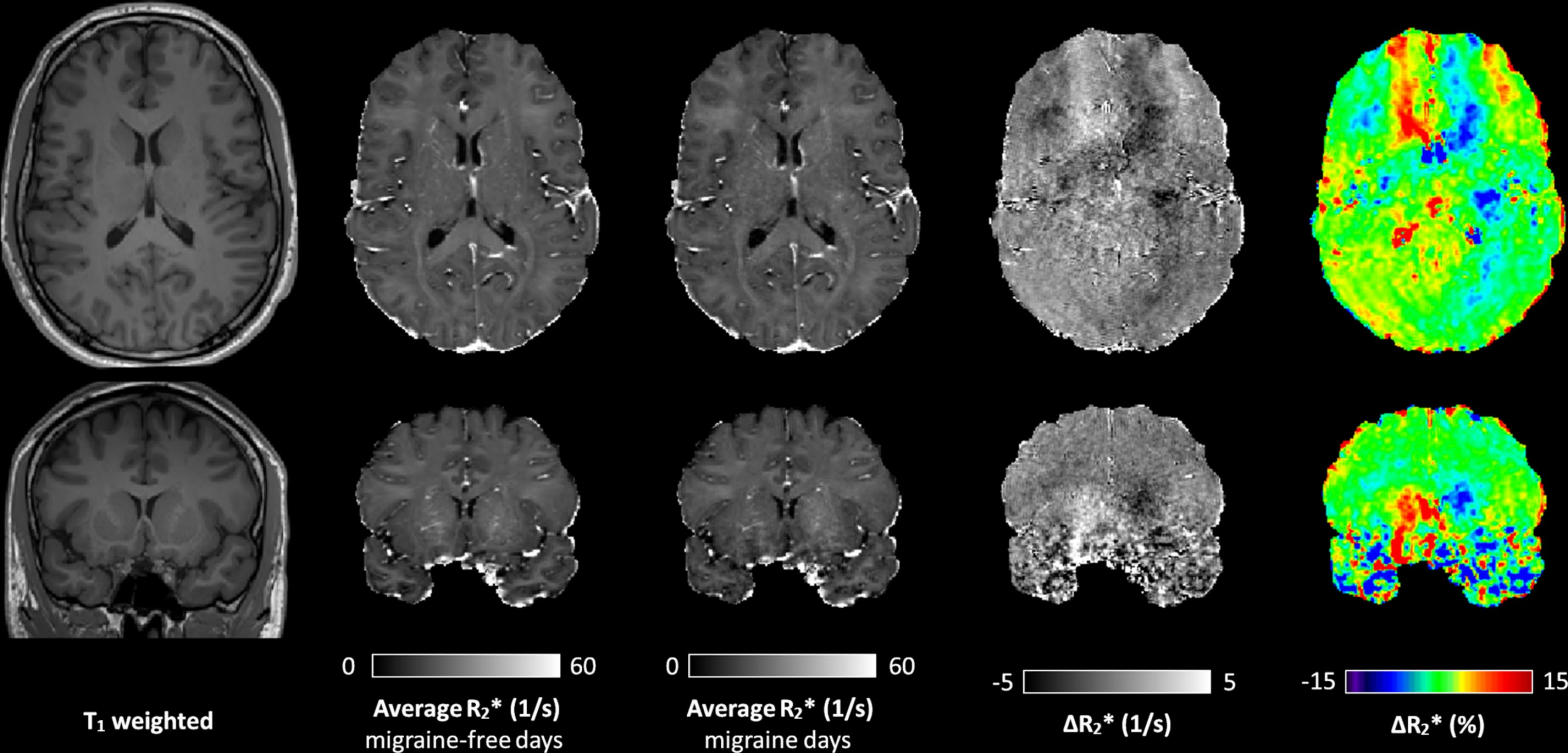
A representative axial (top row) and coronal (bottom row) slice of the *T*_1_ weighted image, average $R_{2}^{*}$ map of all migraine-free days, average $R_{2}^{*}$ map of all migraine days, and the Δ$R_{2}^{*}$ maps between migraine-free and migraine days in absolute values and in percentage, respectively. Δ$R_{2}^{*}$ maps highlight areas with altered $R_{2}^{*}$ during a migraine attack. In the Δ$R_{2}^{*}$ map, an increase in $R_{2}^{*}$ is depicted in red, and a decrease in $R_{2}^{*}$ depicted in blue.

Figure 1 shows representative *T*_1_ weighted images, average $R_{2}^{*}$ maps of all migraine-free days, average $R_{2}^{*}$ maps of all migraine days and the $R_{2}^{*}$ difference (Δ$R_{2}^{*}$ map) between migraine-free and migraine condition of a axial (top row) and coronal (bottom row) slice. The Δ$R_{2}^{*}$ map highlight areas which are altered during a migraine attack, indicating an increase in $R_{2}^{*}$ in red and a decrease in $R_{2}^{*}$ in blue.

### Regional analysis of $R_{2}^{*}$

**Figure 2:**
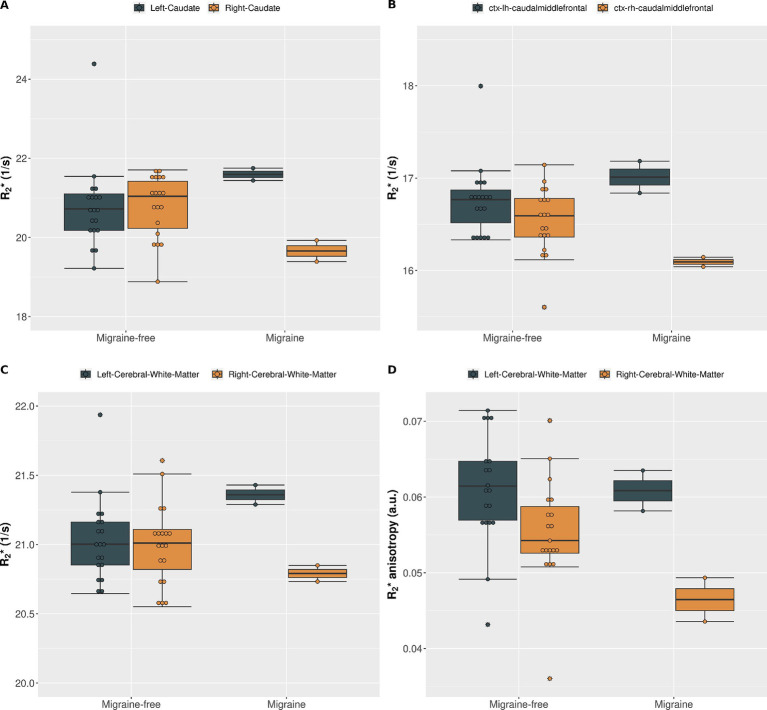
(A) $R_{2}^{*}$ of the left caudate (gray) and right caudate (gold) averaged over all migraine-free days (*n* = 19) and migraine days (*n* = 2). (B) $R_{2}^{*}$ of the left (gray) and right (gold) caudal middle frontal cortex averaged over all migraine-free days (*n* = 19) and migraine days (*n* = 2). (C) $R_{2}^{*}$ of the left (gray) and right (gold) cerebral white matter averaged over all migraine-free days (*n* = 19) and migraine days (*n* = 2). (D) $R_{2}^{*}$ anisotropy of the left (gray) and right (gold) cerebral white matter averaged over all migraine-free days (*n* = 19) and migraine days (*n* = 2).

During migraine attacks, $R_{2}^{*}$ was found to be significantly altered in various brain regions. Overall, an increase in $R_{2}^{*}$ is predominantly observed in brain regions of the left hemisphere, whereas a decrease of $R_{2}^{*}$ is predominantly observed in brain regions of the right hemisphere. In the caudate, $R_{2}^{*}$ increased by 4.9% from 20.6 1/s to 21.6 1/s (*p* = 0.021) in the left hemisphere and decreased by −5.3% from 20.8 1/s to 19.7 1/s (*p* = 0.114) in the right hemisphere, from migraine-free days to during migraine, respectively (Figure 2 and Table 1). These alterations in $R_{2}^{*}$ are also evident in the Δ$R_{2}^{*}$ map shown in Figure 1. $R_{2}^{*}$ increased in the left ventral diencephalon by 5.7% from 23.0 1/s to 24.3 1/s (*p* = 0.011) and left cerebral white matter by 1.9% from 21.0 1/s to 21.4 1/s (*p* = 0.021) on days with migraine. During a migraine attack, $R_{2}^{*}$ decreased in the right superior frontal cortex by −1.9% from 15.6 1/s to 15.3 1/s (*p* = 0.026), in the right caudal middle frontal cortex by −3.0% from 16.6 1/s to 16.1 1/s (*p* = 0.021) (Figure 2) and in the right pericalcarine cortex by −4.6% from 19.7 1/s to 18.8 1/s (*p* = 0.046).

All other structures showed no statistical significant changes in $R_{2}^{*}$ during a migraine attack compared to migraine-free days. A summary of all $R_{2}^{*}$ values in each region and grouped by condition is given in Table 1.

### Anisotropic $R_{2}^{*}$ analysis

**Figure 3:**
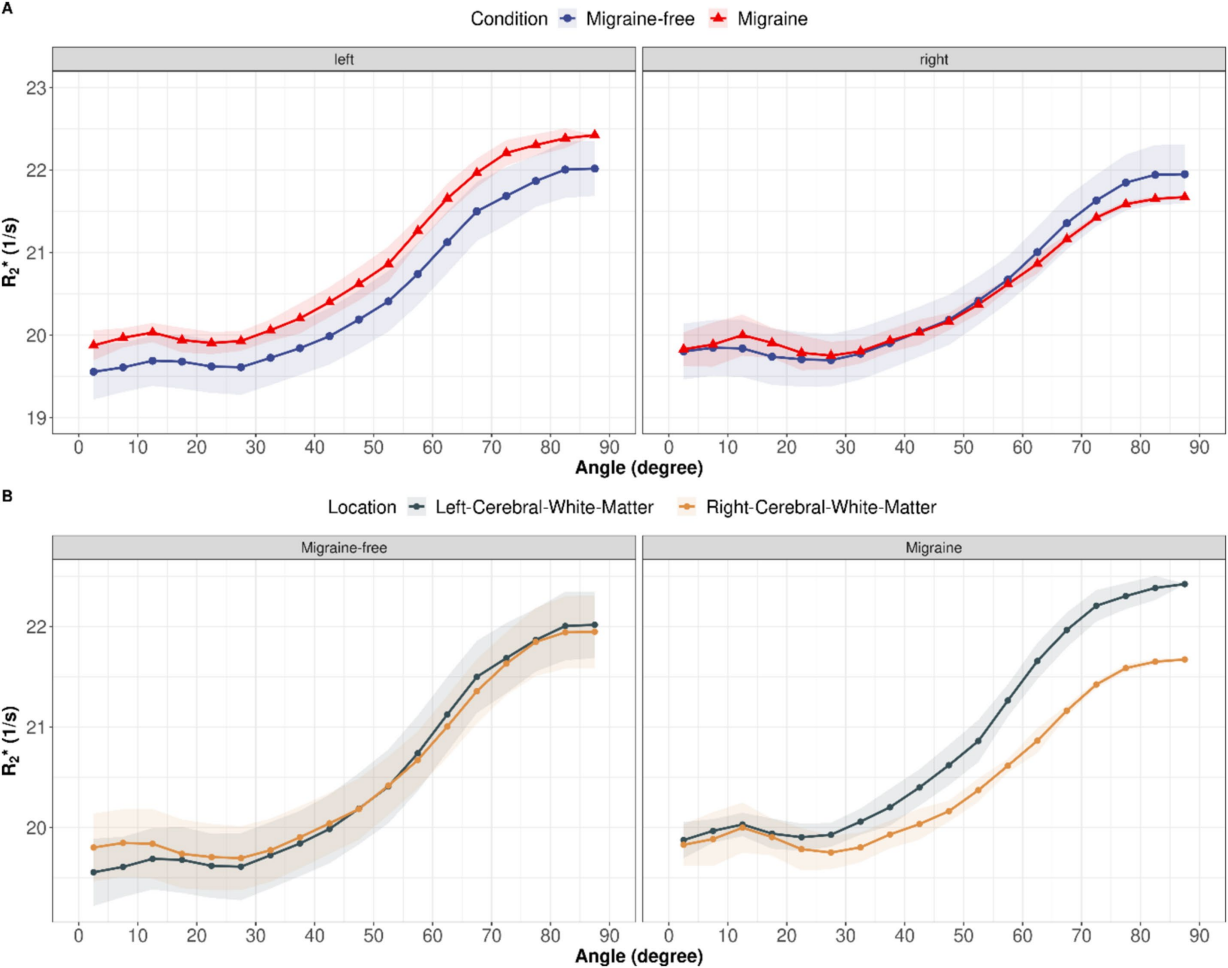
(A) $R_{2}^{*}$ as function of fiber angle for left and right cerebral white matter and averaged over all migraine-free days (blue curves) and days with migraine (red curves). (B) $R_{2}^{*}$ as function of fiber angle split into migraine-free days (left) and migraine days (right). Orientation dependent $R_{2}^{*}$ for left cerebral white mater is depicted in gray and right cerebral white mater in gold.

$R_{2}^{*}$ orientation dependency was assessed to separate isotropic and anisotropic $R_{2}^{*}$ contributions in cerebral white matter at each day. On average, $R_{2}^{*}$ increased with increasing fiber angle from 19.6 ± 0.3 Hz at 0° to 22.1 ± 0.3 Hz at 90° (12.6%, *p* < 0.001) in the left cerebral white matter and from 19.8 ± 0.3 Hz at 0° to 21.9 ± 0.4 Hz at 90° (10.7%, *p* < 0.001) in the right cerebral white mater. Grouping by condition, revealed alterations in isotropic and anisotropic $R_{2}^{*}$ during a migraine attack compared to migraine-free days as shown in Figure 3. In the left cerebral white matter $R_{2}^{*}$ increased by 1.8% (*p* = 0.021) and $R_{2}^{*}$ anisotropy decreased by −1.9% (*p* = 0.853), where as in the right cerebral white matter $R_{2}^{*}$ decreased by −1.0% (*p* = 0.286) and $R_{2}^{*}$ anisotropy decreased by −16.6% (*p* = 0.011). $R_{2}^{*}$ anisotropy differs between left and right cerebral white matter by −9.2% (*p* = 0.009) on migraine-free days and by −23.6% (*p* < 0.001) on days with migraine.

## Discussion

In contrast to the literature, where quantitative MRI in patients with migraine is mainly acquired in cross-sectional studies, we aimed to identify potential short-term changes in quantitative MRI to study tissue composition during the migraine cycle, and in particular during an acute migraine attack. To the best of our knowledge, this is the first study in which quantitative MRI was acquired on multiple consecutive days in a patient with migraine, including days with imaging during an acute migraine attack.

The results of this study suggest that $R_{2}^{*}$ relaxometry is suitable for detecting short-term changes in brain tissue composition that are indicative of central iron involvement during an acute migraine attack. We propose that the observed changes in $R_{2}^{*}$ in deep grey matter and cortical brain regions are related to changes in iron content, whereas in white matter an increase in iron content is accompanied by microstructural changes related to anisotropic tissue components, such as vascular structures. Fluctuations in iron content and anisotropic tissue components during a migraine attack are fully reversible within the time period observed.

### Altered iron content in migraine

Several studies observed higher *R*_2_, $R_{2}^{*}$ or magnetic susceptibility values in patients with migraine compared to healthy controls, indicating an increased iron accumulation in various regions of the brain (22). An increased iron content was mainly observed in deep gray matter and cortical gray matter (6, 7, 11–14, 26). Higher iron content in migraine patients were correlated with disease duration and the frequency of migraine attacks (6, 7, 12, 26). Furthermore, it was shown that iron content in the basal ganglia differs between patients with chronic migraine compared to patients with episodic migraine (11, 14, 26).

Studies conducted by Dominguez et al. (11), and Chen et al. (26) showed that patients with chronic migraine have an increased accumulation of iron in areas involved in the nociceptive network such as the red nucleus and periaqueductal gray (PAG). The role of the PAG as a contributory generator in migraine attacks warrants further investigation since several studies, such as the one conducted by Welch et al. (27), point to its importance in the development of migraine attacks. Welch et al. (27) demonstrated that iron homeostasis in the PAG may be affected by recurrent migraine attacks by observing a significant increase in mean $R_{2}^{\prime }$ (=$R_{2}^{*}$ − *R*_2_) and $R_{2}^{*}$ in patients with both episodic migraine and chronic daily headache, although there was no significant difference between the episodic migraine and chronic daily headache groups. In a recent study, investigated changes in iron deposition after treatment with erenumab showed lower $R_{2}^{*}$ values in the PAG and anterior cingulate cortex (ACC), indicating less iron deposition, in responders compared to non-responders after 8 weeks of treatment (28).

Overall, the majority of studies investigating iron content in migraine indicate a general increased iron accumulation and differences in iron content between migraine types (22). In contrast to literature, our study allowed to investigate short-term dynamic alterations in iron content during the migraine cycle. By daily measuring $R_{2}^{*}$ we could observe both, an increase and a decrease in $R_{2}^{*}$, in various regions of the brain being dependent on migraine attack status. In deep gray matter structures and in the cortex, these $R_{2}^{*}$ alterations are highly likely to be driven by iron changes. These dynamic alterations in $R_{2}^{*}$ differed not only between regions but also between hemispheres, indicating a shift in regional iron content.

An increase in $R_{2}^{*}$ is predominantly observed in the left hemisphere, which was also the hemisphere where the pain was located. This could indicate that in these brain regions a higher demand of energy metabolism, and thus a higher need of iron, is present during a migraine attack (29). The accompanied decrease of $R_{2}^{*}$ in contralateral brain regions could indicate a shift of iron between the hemispheres. However, after the migraine attack, iron content in deep gray matter and white matter, reached the same level as on migraine-free days. Our results indicating a short-term alteration in brain iron levels during a migraine attack do not contradict an overall abnormal long-term iron accumulation in patients with migraine.

### Migraine affects anisotropic $R_{2}^{*}$ in white matter

In white matter the effect of iron on $R_{2}^{*}$ is overshadowed by the effect of diamagnetic myelin (15, 16), and orientation effects of anisotropic tissue components. In white matter, there are two main sources of orientation dependency of $R_{2}^{*}$: (I) the orientation of myelinated white matter fibers with respect to *B*_0_ (17, 18) and (II) the anisotropic part of the vasculature (30). Blood vessels have an isotropic component (capillary bed) and an anisotropic component (larger vessels). There is evidence that these larger blood vessels in white matter converge in parallel to main white matter fiber tracts and therefore contribute to the orientation dependent MR signal (31–34). Therefore, we acquired orientation dependent $R_{2}^{*}$ to differentiate between isotropic effects of iron and anisotropy effects of myelin and vascular components contributing to $R_{2}^{*}$ in cerebral white matter.

By separating isotropic and anisotropic contributions to $R_{2}^{*}$, it was possible to identify an increase in white mater iron content on days with migraine. To the best of our knowledge, this study is the first of its kind, which reported alterations in white matter iron content in patients with migraine. Our results could potentially indicate a shift in iron content from deep gray matter structures to white mater during an acute migraine attack.

Beside changes in iron content, migraine is also associated with changes in vascular structures, including veins and perivascular spaces (PVS) (22). Breiding et al. (35) observed a higher total cerebral vein volume in patients with migraine compared to healthy controls. Furthermore, in patients with unilateral migraine, the veins were more prominent in one hemisphere (35). This is in line with our observation of more prominent venous structures in the left hemisphere of our patient. We observed that $R_{2}^{*}$ anisotropy is approximately 10% higher in the left hemisphere compared to the right hemisphere on migraine-free days, indicating higher venous and or PVS volume. On days with migraine, the difference in $R_{2}^{*}$ anisotropy between left and right hemisphere increased up to approximately 30%. This indicates an involvement of vascular mechanism’s during a migraine attack in addition to a slight increase in iron content in cerebral white matter. The decrease in $R_{2}^{*}$ anisotropy during migraine could be explained by a lower venous volume, or altered perfusion which is commonly observed in migraine (36, 37) or by a reduction of the PVS volume. A closure of the PVS causing an impaired glymphatic flow during migraine was observed in a previous study (38). Studies investigating PVS in patients with migraine showed inconclusive results where both increased and decreased, PVS volume was observed compared to healthy controls (38–41). Overall, the majority of the studies indicated an increase in PVS volume. This would be inline with the observation of enlarged PVS in our patient. However, an overall higher PVS volume in patients with migraine does not contradict a decrease in PVS volume during an acute migraine attack.

Altered mean arterial blood pressure can induce cerebral blood volume shifts, detectable through quantitative susceptibility mapping (QSM) (42). Changes in blood volume or deoxygenated hemoglobin concentration during acute migraines may decrease $R_{2}^{*}$ anisotropy, recovering post-attack. Besides vascular factors, $R_{2}^{*}$ anisotropy reflects axonal and myelin-related alterations. Granziera et al. (13) noted thalamic changes in migraine patients, including myelin and cellularity differences, along with iron content changes. Palm-Meinders et al. (6) observed elevated *R*_2_ values in migraine patients initially, which were decreasing after 9 years. Thus, migraine-related iron changes might be obscured by age or disease-related tissue changes. Numerous studies used DTI to assess white matter alterations, showing heterogeneous results in migraine (43).

Alterations of tissue microstructure in patients with migraine will clearly affect $R_{2}^{*}$ anisotropy in general. However, we observed that after migraine attacks, the altered $R_{2}^{*}$ anisotropy values reach the same level as on migraine-free days. These short-term alterations in $R_{2}^{*}$ anisotropy are more likely to be explained by vascular effects, rather than microstructural tissue changes linked to myelin, as dominant source of $R_{2}^{*}$ anisotropy.

To investigate if there are brain changes on migraine-free days right after the migraine attack, we repeated the entire analysis, excluding these days. We did not observe any change in our overall results and present a summary of all results without days right after the migraine attack as Supplementary material.

### Limitations

Although our results are solely based on a single patient, this study is the first-of-its-kind acquiring MRI on multiple consecutive days, comprising migraine-free days and days with acute migraine attacks. Acquiring MRI of multiple patients with migraine on 21 or more days, including days with a migraine attack, would be very challenging. It is worth to note that is extremely difficult to recruit patients which are willing to undergo an MRI examination during an acute migraine attack, especially if a refrain from taking any preventive of acute medication is required for study purpose. Furthermore, results of multiple subject could not directly be averaged, as multiple factors, such as unilateral or bilateral migraine location of the pain, duration and frequency of the migraine attacks, age and sex will influence the observed $R_{2}^{*}$ alterations.

However, further multi-parametric MRI studies with a larger number of patients, ideally with image acquisition during migraine attacks, will be needed to further identify the cellular mechanism contributing to isotropic and anisotropic $R_{2}^{*}$ changes in migraine and to obtain reproducible results. Furthermore, future studies should also consider the severity of the disease, e.g., attack frequency and duration. Moreover, the exact onset and the end of the migraine attack cannot always be assessed with high accuracy due to clinical considerations such as sleep terminating the migraine attack.

In our study, we observed a relative short time period of 21 days in comparison to years of disease duration, thus no conclusions about long-term alterations in iron content or vascular structures can be made from our results. Although, our results indicate that changes in iron content related to migraine attacks are reversible, a long-term alteration in iron content can still accompany short-term dynamic fluctuations in iron content.

It is worth to note, that plotting of $R_{2}^{*}$ on each day in combination with the boxplots is important, as every day was assigned to only one condition (e.g., migraine-free or migraine), yet, as shown in Figure 2A, altered $R_{2}^{*}$ values can sometimes be observed already on days prior or after the migraine attack. This can lead to a bias in the boxplots and statistical analysis. In addition, potential outliers, as shown in Figure 2A can be identified by combining day plots with boxplots.

While $R_{2}^{*}$ is highly sensitive to iron content, it can not be directly converted to an absolute iron concentration. Although, studies have shown a strong correlation between $R_{2}^{*}$ values and iron concentration in brain tissue, particularly in deep gray matter structures (44), the exact relationship is not perfectly linear and can vary across brain regions. An approximation of the altered iron content can be made based on a post-mortem study, where $R_{2}^{*}$ was validated as reliable measure for iron content using mass spectrometry (10). Langkammer et al. (10) reported a slope of 0.27 1/s per mg/kg iron for gray matter. Based on this study, the $R_{2}^{*}$ decrease of around 5% in the caudate would correspond to a change in iron content of approximately 3.7 mg/kg wet tissue.

## Conclusion

We conclude that migraine attacks lead to short-term changes in $R_{2}^{*}$ in specific brain regions, which further differ between the left and right hemispheres. Our study identified both specific brain regions with increased and brain regions with decreased iron content during a migraine attack. This suggests that different metabolic processes have an increased need for iron, which could potentially be resolved by shifting iron between brain structures. Furthermore, by separating isotropic and anisotropic $R_{2}^{*}$ components, we were able to distinguish between iron and non-iron related tissue changes in the cerebral white matter. Our observed decrease in $R_{2}^{*}$ anisotropy during a migraine attack suggests the involvement of vascular components, such as a decrease in PVS volume, a change in venous volume, or a blood pressure-induced shift in magnetic susceptibility during an acute migraine attack. However, the observed $R_{2}^{*}$ changes fully return to baseline after the migraine attack has resolved. This supports the involvement of vascular structures rather than changes in axonal fibre architecture and myelin content as the dominant source of $R_{2}^{*}$ anisotropy. In conclusion, the time-dependent mapping of $R_{2}^{*}$ during a migraine cycle opens new possibilities to study short-term changes in the brain during a migraine attack, which appear to be partially different from long-term tissue changes in migraineurs. Taken together, our results indicate dynamic alterations in iron metabolism and vascular processes during an acute migraine attack.

## Data Availability

The data that support the findings of this study are available on request from the corresponding author (VF). The data are not publicly available as they contain information that could compromise the privacy of the research participant.
