# Supplementary material for: Dynamic fluctuations in brain iron content during migraine attacks: insights from relaxometry and diffusion tensor imaging
Source: Front Neurol. 2024 Dec 20;15:1422313. doi: 10.3389/fneur.2024.1422313 (PMC11697585; doi:10.3389/fneur.2024.1422313)
Supplement: Supplementary file 1 [file Data_sheet_1.DOCX]

**Supplementary results**

Excluding days right after the migraine attack from the migraine-free days.

#### Regional analysis of R2*

During migraine attacks, R2* was found to be significantly altered in various brain regions. Overall, an increase in R2* is predominantly observed in brain regions of the left hemisphere, whereas a decrease of R2* is predominantly observed in brain regions of the right hemisphere. In the caudate, R2* increased by 5.0% from 20.5 1/s to 21.6 1/s (p = 0.03) in the left hemisphere and decreased by -5.8% from 20.8 1/s to 19.7 1/s (p = 0.11) in the right hemisphere, from migraine-free days to during migraine, respectively (Supplementary Figure 2 and Supplementary Table 1). These alterations in R2* are also evident in the ΔR2* map shown in Supplementary Figure 1. R2* increased in the left ventral diencephalon by 5.3% from 23.0 1/s to 24.3 1/s (p = 0.01) and left cerebral white matter by 1.9% from 21.0 1/s to 21.4 1/s (p = 0.03) on days with migraine. During a migraine attack, R2* decreased in the right superiorfrontal cortex by -2.0% from 15.6 1/s to 15.3 1/s (p = 0.03), in the right caudalmiddlefrontal cortex by -3.1% from 16.6 1/s to 16.1 1/s (p = 0.03) (Figure 2) and in the right pericalcarine cortex by -4.5% from 19.6 1/s to 18.8 1/s (p = 0.01). In addition, R2* decreased by -5.1% from 24.3 1/s to 23.1 1/s (p = 0.02) in the posterior corpus callosum and by -4.9% from 20.2 1/s to 19.3 1/s (p = 0.03) in the right cerebellum cortex.

All other structures showed no statistical significant changes in R2* during a migraine attack compared to migraine-free days. A summary of all R2* values in each region and grouped by condition is given in Table 1.

#### Anisotropic R2* analysis

R2* orientation dependency was assessed to separate isotropic and anisotropic R2* contributions in cerebral white matter at each day. On average, R2* increased with increasing fiber angle from 19.6 ± 0.3 Hz at 0° to 22.1 ± 0.3 Hz at 90° (12.9%, p < 0.001) in the left cerebral white matter and from 19.8 ± 0.3 Hz at 0° to 21.9 ± 0.4 Hz at 90° (10.6%, p < 0.001) in the right cerebral white mater. Grouping by condition, revealed alterations in isotropic and anisotropic R2* during a migraine attack compared to migraine-free days as shown in Figure 3. In the left cerebral white matter R2* increased by 1.9% (p = 0.03) and R2* anisotropy decreased by -2.0% (p = 0.84), where as in the right cerebral white matter R2* decreased by -0.9% (p = 0.26) and R2* anisotropy decreased by -15.6% (p = 0.04). R2* anisotropy differs between left and right cerebral white matter by -11.4% (p = 0.002) on migraine-free days and by -23.6% (p < 0.001) on days with migraine.


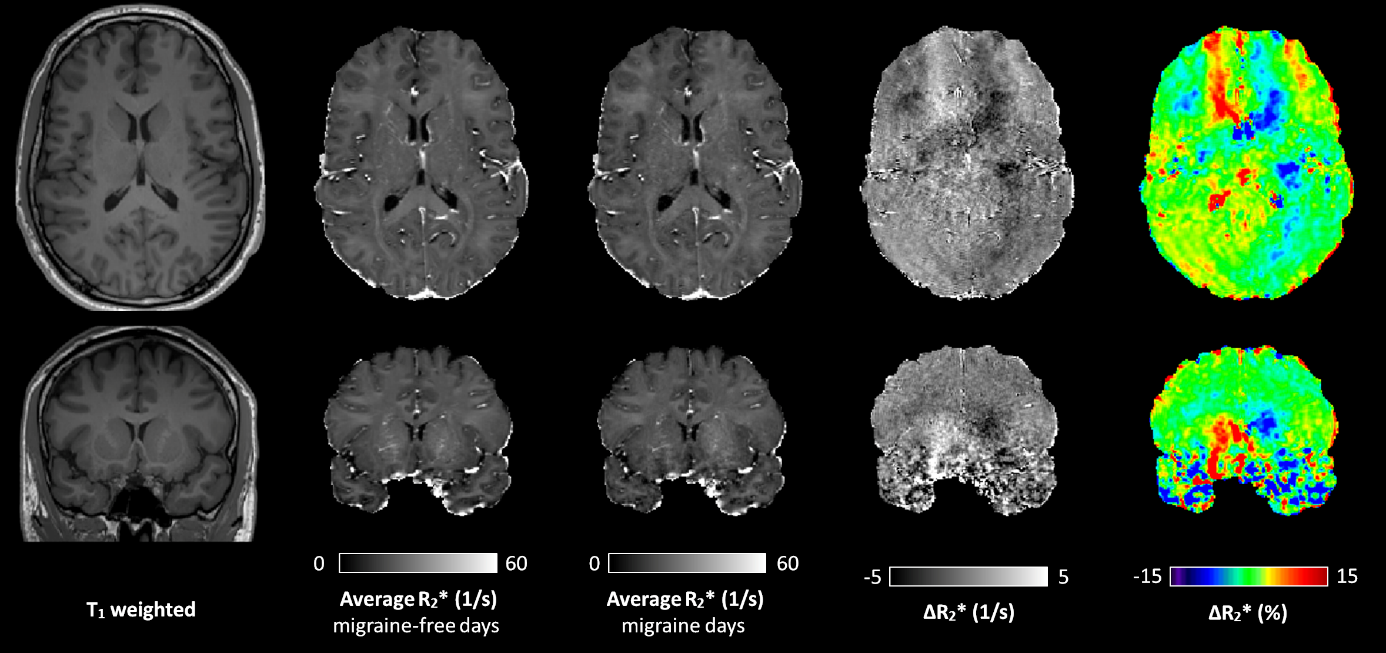


**Supplementary Figure 1:** A representative axial (top row) and coronal (bottom row) slice of the T1 weighted image, average R2* map of all migraine-free days (excluding the day right after the migraine attack) and migraine days, and the ΔR2* between migraine-free and migraine days, highlighting areas with altered R2* during a migraine attack. In the ΔR2* map, an increase in R2* is depicted in red, and a decrease in R2* depicted in blue.


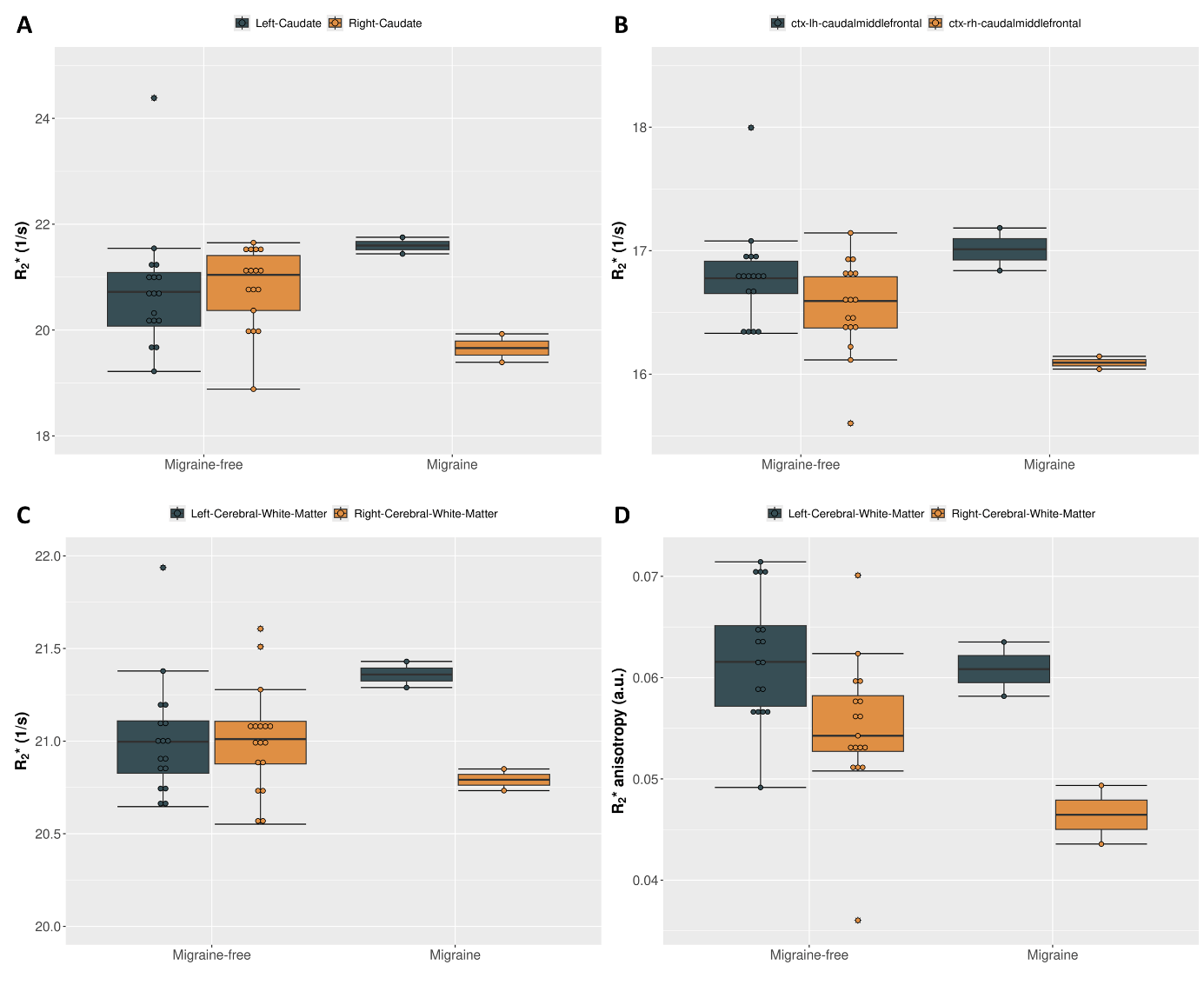


**Supplementary Figure 2:** (A) R2* of the left caudate (gray) and right caudate (gold) averaged over all migraine-free days (n = 17) and migraine days (n = 2). (B) R2* of the left (gray) and right (gold) caudalmiddlefrontal cortex averaged over all migraine-free days (n = 17) and migraine days (n = 2). (C) R2* of the left (gray) and right (gold) cerebral white matter averaged over all migraine-free days (n = 17) and migraine days (n = 2). (D) R2* anisotropy of the left (gray) and right (gold) cerebral white matter averaged over all migraine-free days (n = 17) and migraine days (n = 2). Days right after the migraine attack are excluded from the migraine free days.


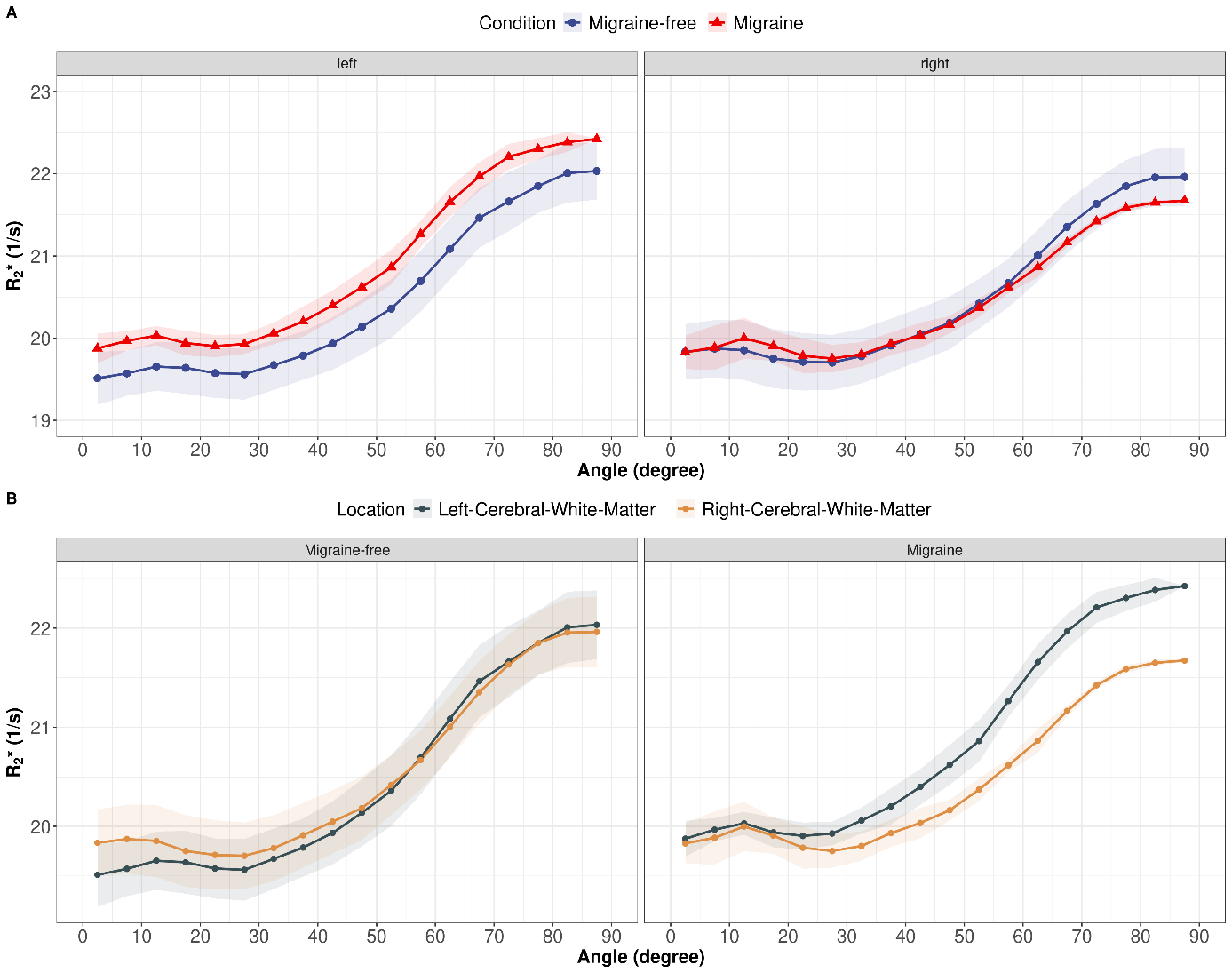


**Supplementary Figure 3:** (A) R2* as function of fiber angle for left and right cerebral white matter and averaged over all migraine-free days (blue curves) and days with migraine (red curves). (B) R2* as function of fiber angle split into migraine-free days (left) and migraine days (right). Orientation dependent R2* for left cerebral white mater is depicted in gray and right cerebral white mater in gold. Days right after the migraine attack are excluded from the migraine free days.

**Supplementary Table 1:** Regional R_2_* values averaged over all migraine-free days (excluding days right after the migraine attack) and migraine days. R2* difference between migraine-free and migraine condition is given in percentage.

|  | **Hemisphere** | **R2* (1/s)** | | | | **delta R2* (%)** | **P-value** |  |
| --- | --- | --- | --- | --- | --- | --- | --- | --- |
|  |  | **Migraine-free** | | **Migraine** | |  |  |  |
|  |  | mean | SD | mean | SD |  |  |  |
| Thalamus | left | 20.6 | 0.5 | 20.6 | 0.1 | -0.2 | 0.84 |  |
|  | right | 20.7 | 0.5 | 20.4 | 0.5 | -1.3 | 0.49 |  |
| Caudate | left | 20.5 | 0.7 | 21.6 | 0.2 | 5.0 | **0.03** |  |
|  | right | 20.8 | 0.8 | 19.7 | 0.4 | -5.8 | 0.11 |  |
| Putamen | left | 23.2 | 0.4 | 23.0 | 0.8 | -1.0 | 0.57 |  |
|  | right | 22.6 | 0.6 | 22.9 | 0.1 | 1.4 | 0.42 |  |
| Pallidum | left | 31.4 | 1.1 | 32.0 | 0.6 | 1.7 | 0.49 |  |
|  | right | 31.0 | 0.7 | 31.6 | 0.1 | 4.1 | 0.05 |  |
| Hippocampus | left | 17.0 | 0.4 | 17.7 | 0.1 | 3.5 | 0.08 |  |
|  | right | 17.1 | 0.4 | 16.8 | 0.5 | -1.8 | 0.47 |  |
| Amygdala | left | 16.0 | 0.6 | 15.2 | 0.1 | -5.2 | 0.13 |  |
|  | right | 14.8 | 0.7 | 14.8 | 0.7 | 0.0 | 0.99 |  |
| Accumbens-area | left | 17.6 | 1.4 | 16.9 | 1.8 | -4.1 | 0.49 |  |
|  | right | 18.3 | 1.0 | 17.2 | 0.4 | -6.3 | 0.23 |  |
| VentralDC | left | 23.0 | 0.5 | 24.3 | 0.3 | 5.3 | **0.01** |  |
|  | right | 22.1 | 0.7 | 21.0 | 0.5 | -5.5 | 0.07 |  |
| Cerebral-White-Matter | left | 21.0 | 0.2 | 21.4 | 0.1 | 1.9 | **0.03** |  |
|  | right | 21.0 | 0.3 | 20.8 | 0.1 | -0.9 | 0.26 |  |
| Optic-Chiasm |  | 18.8 | 1.9 | 18.0 | 0.7 | -4.5 | 0.55 |  |
| CC-Posterior |  | 24.3 | 0.7 | 23.1 | 0.3 | -5.1 | **0.02** |  |
| CC-Mid-Posterior |  | 20.6 | 0.9 | 20.5 | 0.2 | -0.3 | 0.66 |  |
| CC-Central |  | 19.7 | 1.0 | 20.9 | 0.1 | 5.8 | 0.19 |  |
| CC-Mid-Anterior |  | 18.0 | 0.6 | 18.1 | 0.3 | 0.3 | 0.99 |  |
| CC-Anterior |  | 22.0 | 1.2 | 20.9 | 0.6 | -5.5 | 0.20 |  |
| Bankssts | left | 18.1 | 0.4 | 18.2 | 0.1 | 0.8 | 0.84 |  |
|  | right | 17.8 | 0.4 | 17.2 | 0.4 | -3.4 | 0.08 |  |
| caudalanteriorcingulate | left | 14.9 | 1.2 | 15.6 | 0.5 | 4.7 | 0.35 |  |
|  | right | 14.9 | 0.9 | 14.8 | 0.1 | -0.5 | 0.57 |  |
| caudalmiddlefrontal | left | 16.7 | 0.3 | 17.0 | 0.2 | 1.7 | 0.12 |  |
|  | right | 16.6 | 0.3 | 16.1 | 0.1 | -3.1 | **0.03** |  |
| cuneus | left | 21.3 | 0.7 | 21.0 | 0.6 | -1.5 | 0.57 |  |
|  | right | 23.4 | 0.8 | 23.5 | 0.1 | 0.6 | 0.94 |  |
| entorhinal | left | 22.2 | 2.7 | 22.5 | 1.8 | 1.4 | 0.84 |  |
|  | right | 18.6 | 2.2 | 19.7 | 0.2 | 5.4 | 0.84 |  |
| fusiform | left | 21.0 | 0.6 | 21.6 | 0.5 | 2.6 | 0.27 |  |
|  | right | 20.5 | 0.5 | 20.1 | 0.1 | -1.8 | 0.12 |  |
| inferiorparietal | left | 17.6 | 0.4 | 17.7 | 0.5 | 0.6 | 0.75 |  |
|  | right | 18.1 | 0.4 | 17.3 | 0.5 | -4.9 | 0.07 |  |
| inferiortemporal | left | 24.4 | 0.8 | 24.7 | 0.2 | 1.3 | 0.73 |  |
|  | right | 24.3 | 1.1 | 24.4 | 0.5 | 0.6 | 0.99 |  |
| isthmuscingulate | left | 19.8 | 0.5 | 19.3 | 0.1 | -2.5 | 0.26 |  |
|  | right | 19.4 | 0.9 | 18.4 | 0.2 | -5.3 | 0.07 |  |
| lateraloccipital | left | 20.8 | 0.4 | 20.5 | 0.2 | -1.2 | 0.35 |  |
|  | right | 20.8 | 0.5 | 20.0 | 0.5 | -3.8 | 0.05 |  |
| lateralorbitofrontal | left | 27.5 | 0.9 | 27.7 | 0.1 | 0.9 | 0.57 |  |
|  | right | 27.3 | 0.7 | 26.6 | 0.6 | -2.5 | 0.21 |  |
| lingual | left | 20.3 | 0.7 | 20.1 | 0.2 | -1.2 | 0.84 |  |
|  | right | 21.5 | 0.7 | 20.7 | 0.2 | -4.0 | 0.11 |  |
| medialorbitofrontal | left | 23.2 | 0.8 | 23.8 | 1.0 | 2.4 | 0.47 |  |
|  | right | 21.4 | 1.2 | 20.2 | 1.1 | -6.0 | 0.19 |  |
| middletemporal | left | 18.2 | 0.5 | 18.0 | 0.2 | -1.2 | 0.57 |  |
|  | right | 19.6 | 0.6 | 19.5 | 0.2 | -0.4 | 0.84 |  |
| parahippocampal | left | 25.2 | 0.9 | 25.5 | 1.0 | 1.1 | 0.64 |  |
|  | right | 19.6 | 0.8 | 19.1 | 0.8 | -2.4 | 0.42 |  |
| paracentral | left | 18.0 | 0.4 | 18.3 | 0.1 | 1.5 | 0.29 |  |
|  | right | 17.9 | 0.4 | 18.1 | 0.1 | 1.2 | 0.47 |  |
| parsopercularis | left | 17.0 | 0.2 | 16.6 | 0.1 | -2.2 | 0.05 |  |
|  | right | 15.9 | 0.4 | 15.5 | 0.1 | -2.7 | 0.19 |  |
| parsorbitalis | left | 18.5 | 0.6 | 18.0 | 0.9 | -2.9 | 0.35 |  |
|  | right | 18.3 | 0.6 | 18.5 | 0.2 | 1.1 | 0.49 |  |
| parstriangularis | left | 17.3 | 0.5 | 16.9 | 0.2 | -2.2 | 0.14 |  |
|  | right | 16.8 | 0.5 | 16.6 | 0.2 | -1.2 | 0.75 |  |
| pericalcarine | left | 19.8 | 0.6 | 19.9 | 0.2 | 0.3 | 0.75 |  |
|  | right | 19.6 | 0.4 | 18.8 | 0.1 | -4.5 | **0.01** |  |
| postcentral | left | 18.0 | 0.2 | 17.9 | 0.2 | -0.6 | 0.39 |  |
|  | right | 17.6 | 0.4 | 17.3 | 0.1 | -1.6 | 0.35 |  |
| posteriorcingulate | left | 16.5 | 0.3 | 16.3 | 0.1 | -1.1 | 0.35 |  |
|  | right | 16.4 | 0.1 | 16.4 | 0.1 | 0.2 | 0.75 |  |
| precentral | left | 17.8 | 0.3 | 17.7 | 0.3 | -0.5 | 0.95 |  |
|  | right | 17.6 | 0.3 | 17.3 | 0.1 | -1.8 | 0.13 |  |
| precuneus | left | 18.0 | 0.4 | 18.1 | 0.3 | 0.8 | 0.49 |  |
|  | right | 17.8 | 0.5 | 17.4 | 0.4 | -2.1 | 0.35 |  |
| rostralanteriorcingulate | left | 15.1 | 1.2 | 15.2 | 0.1 | 0.8 | 0.66 |  |
|  | right | 14.4 | 1.0 | 13.8 | 0.3 | -4.0 | 0.42 |  |
| rostralmiddlefrontal | left | 17.3 | 0.3 | 16.9 | 0.3 | -2.2 | 0.14 |  |
|  | right | 17.4 | 0.3 | 17.3 | 0.2 | -0.6 | 0.75 |  |
| superiorfrontal | left | 15.7 | 0.5 | 16.1 | 0.2 | 2.3 | 0.39 |  |
|  | right | 15.6 | 0.2 | 15.3 | 0.1 | -2.0 | **0.03** |  |
| superiorparietal | left | 18.1 | 0.3 | 17.9 | 0.3 | -0.9 | 0.49 |  |
|  | right | 18.1 | 0.4 | 17.8 | 0.4 | -1.7 | 0.29 |  |
| superiortemporal | left | 16.8 | 0.3 | 16.9 | 0.1 | 0.9 | 0.55 |  |
|  | right | 16.2 | 0.2 | 16.1 | 0.3 | -0.7 | 0.47 |  |
| supramarginal | left | 17.5 | 0.3 | 17.7 | 0.2 | 1.2 | 0.33 |  |
|  | right | 17.3 | 0.3 | 17.0 | 0.5 | -1.9 | 0.33 |  |
| frontalpole | left | 16.9 | 1.2 | 16.3 | 0.5 | -3.9 | 0.35 |  |
|  | right | 16.3 | 0.9 | 16.1 | 0.7 | -1.2 | 0.95 |  |
| temporalpole | left | 15.2 | 1.6 | 16.1 | 0.5 | 5.6 | 0.49 |  |
|  | right | 14.1 | 1.6 | 14.8 | 0.5 | 5.0 | 0.66 |  |
| transversetemporal | left | 18.1 | 0.6 | 18.6 | 0.2 | 3.0 | 0.16 |  |
|  | right | 17.0 | 0.4 | 16.7 | 0.3 | -1.5 | 0.47 |  |
| insula | left | 16.2 | 0.7 | 16.3 | 0.8 | 0.6 | 0.99 |  |
|  | right | 15.7 | 0.4 | 16.1 | 0.3 | 2.4 | 0.33 |  |
| Cerebellum-White-Matter | left | 19.8 | 0.7 | 21.1 | 0.7 | 6.3 | 0.05 |  |
|  | right | 19.9 | 0.9 | 20.4 | 0.1 | 2.3 | 0.42 |  |
| Cerebellum-Cortex | left | 20.3 | 0.6 | 20.2 | 0.1 | -0.5 | 0.99 |  |
|  | right | 20.2 | 0.5 | 19.3 | 0.3 | -4.9 | **0.03** |  |
